# An integrated analysis of the anticarcinogenic role of forkhead box protein 1 in oesophageal squamous cell carcinoma

**DOI:** 10.1111/jcmm.18294

**Published:** 2024-04-23

**Authors:** Guanzhi Ye, Gaojian Pan, Xiaolei Zhu, Ning Li, Hongming Liu, Guojun Geng, Jie Jiang

**Affiliations:** ^1^ Department of Thoracic Surgery The First Affiliated Hospital of Xiamen University Xiamen Fujian China

**Keywords:** expression, FOXP1, oesophageal squamous cell carcinoma, survival prognosis, tumour immune microenvironment

## Abstract

Forkhead box protein 1 (FOXP1) serves as a tumour promoter or suppressor depending on different cancers, but its effect in oesophageal squamous cell carcinoma has not been fully elucidated. This study investigated the role of FOXP1 in oesophageal squamous cell carcinoma through bioinformatics analysis and experimental verification. We determined through public databases that FOXP1 expresses low in oesophageal squamous cell carcinoma compared with normal tissues, while high expression of FOXP1 indicates a better prognosis. We identified potential target genes regulated by FOXP1, and explored the potential biological processes and signalling pathways involved in FOXP1 in oesophageal squamous cell carcinoma through GO and KEGG enrichment, gene co‐expression analysis, and protein interaction network construction. We also analysed the correlation between FOXP1 and tumour immune infiltration levels. We further validated the inhibitory effect of FOXP1 on the proliferation of oesophageal squamous cell carcinoma cells through CCK‐8, colony formation and subcutaneous tumour formation assays. This study revealed the anticarcinogenic effect of FOXP1 in oesophageal squamous cell carcinoma, which may serve as a novel biological target for the treatment of tumour.

## 1 INTRODUCTION

Oesophageal cancer is a highly invasive malignant tumour that contributes significantly to cancer‐related deaths with over 600,000 new cases and nearly 550,000 deaths globally in 2020.^1^ Oesophageal squamous cell carcinoma is the most prevalent type. Patients are usually diagnosed with oesophageal cancer in the middle to late stage of the tumour and lose the opportunity for surgical resection, resulting in a poor prognosis.^2^ The pathogenesis and progression of oesophageal squamous cell carcinoma are still not fully understood, which makes treatment difficult even with recent advancements in surgery, radiation, chemotherapy and immunotherapy. Thus, there is substantial clinical value to a fuller investigation of the molecular mechanisms of oesophageal squamous cell carcinoma. This contributes to a better understanding of biological process of tumour progression and lays the groundwork for the creation of novel therapeutic strategies.

The forkhead box (FOX) proteins are a class of transcription factors widely present from yeast to humans, belonging to a subgroup of ‘helix‐turn‐helix’ proteins. Weigel et al.^3^ first discovered that FOX proteins play an important role in the development of embryos. Further researches have been discovered that FOX protein, as a large and widely functional transcription factor family, involves various biological processes, such as embryonic development, carbohydrate and lipid metabolism, biological ageing, cell differentiation, apoptosis and immune regulation. Its mutations and expression abnormalities are related to developmental malformation, metabolic diseases and tumorigenesis.^4^, ^5^, ^6^ Forkhead box P1 (FOXP1) is an important member of the FOX family, which widely expresses in human normal and tumour tissues to varying degrees. The FOXP1 gene is located in the 3p14.1 region of chromosome, with a length of 628 kb and its complete coding product is a protein containing 677 amino acids.^7^ FOXP1 is reported to be related to the survival prognosis of various tumour patients and it plays a crucial role in the occurrence and development of breast,^8^ ovarian,^9^, ^10^ endometrial,^11^ bladder,^12^, ^13^ prostate,^14^, ^15^ lymphoma,^16^ liver,^17^ colorectal,^18^ gastric,^19^ lung cancer^20^, ^21^ and other tumours. Interestingly, FOXP1 plays a role in promoting or inhibiting cancer in different tumours.

The molecular mechanism of FOXP1 in oesophageal squamous cell carcinoma has not been fully elucidated yet. This study investigated the differential expression of FOXP1 in oesophageal squamous cell carcinoma and its impact on the prognosis of tumour patients through bioinformatics analysis, including gene co‐expression analysis, protein–protein interaction (PPI) network construction, gene ontology (GO) and Kyoto Encyclopedia of Genes and Genomes (KEGG) enrichment, and tumour immune infiltration analysis. The experiments such as Cell Counting Kit‐8 (CCK‐8) assay, colony formation assay, and subcutaneous tumour formation assay were conducted to verify the anti‐tumour effect of FOXP1 in oesophageal squamous cell carcinoma and the signalling pathways involved.

## 2 MATERIALS AND METHODS

### 2.1 Gene expression analysis

We analysed the differential expression level of FOXP1 in various tumour tissues, including oesophageal cancer, compared with normal tissues using the ‘General’ module of the Gene expression profiling interactive analysis (GEPIA) database (http://gepia.cancer‐pku.cn/).^22^ We used the ‘TCGA gene expression’ module of the UALCAN database (https://ualcan.path.uab.edu/) to draw Box plot graphs and analysed the expression differences of FOXP1 depends on different pathological types of oesophageal cancer, tumour grades and tumour stages.^23^, ^24^ Moreover, we determined the differences of FOXP1 mRNA expression level in oesophageal squamous cell carcinoma and normal adjacent tissues using the GSE17351, GSE70409, GSE77861 and GSE100942 datasets from the GEO database (https://www.ncbi.nlm.nih.gov/geo/). We use the ‘violinplot’ and ‘Matplotlib’ functions in the ‘Seaborn’ package in Python to draw violin plots.

### 2.2 Survival prognosis analysis

Tumour Immune Single‐cell Hub 2 (TISCH2) (http://tisch.comp‐genomics.org/) is a single cell RNA sequencing database, which provides cell‐type annotation at the single cell level in various tumors.^25^ We analysed the correlation between the FOXP1 expression and overall survival in different tumour patients, including oesophageal cancer patients, using the ‘Gene’ and ‘TCGA survival’ modules of TISCH2 database. Meanwhile, we investigated the correlation between FOXP1 expression and survival prognosis in oesophageal cancer patients using the ‘Survival’ module of the GEPIA database and KM plotter (http://www.kmplot.com/) database. We further validated the relationship between the FOXP1 mRNA expression and survival prognosis of oesophageal squamous cell carcinoma patients using the GSE53625 dataset in the GEO database. The survival curve was drawn using the ‘lifelines.KaplanMeierFitter’ function in ‘Lifelines’ package in Python.

### 2.3 Identification of differentially expressed genes in high‐ and low‐expression of FOXP1 cohorts

We compared the differentially expressed genes of High‐ and Low‐expression of FOXP1 cohorts using ‘Limma’ package of R software (version 3.40.2) in the GSE26886 and GSE45168 datasets in the GEO database. The differentially expressed genes which met the following criteria were considered significant: adjusted *p* value <0.05 and log_2_ |fold change|>1. The volcano plots and cluster heatmaps were drawn using ‘ggplot2’ and ‘pheatmap’ packages in the R software, respectively.

### 2.4 GO and KEGG enrichment, gene co‐expression analysis, and PPI network construction

The Metascape database (https://metascape.org/) is a gene function annotation tool that integrates multiple data resources such as GO, KEGG, STRING, UniProt and DrugBank, which enables to perform pathway enrichment and biological process annotation.^26^ We conducted GO and KEGG enrichment analysis on differentially expressed genes using ‘Matplotlib’ and ‘Plotly’ packages in Python to explore the biological functions and signalling pathways. Moreover, we conducted GO enrichment analysis through the Metascape database. The GO cord graph was drawn using the ‘circlize’ and ‘plotly’ packages in the R software. The gene co‐expression analysis of FOXP1 was performed using the GeneMANIA database (http://genemania.org/), including datasets and interaction information from GEO, BioGRID, IRefIndex and I2D databases.^26^ The PPI network for differentially expressed genes was conducted using the STRING database (https://string‐db.org/) and the important MCODE modules were determined using the Metascape database.^27^

### 2.5 Immunohistochemistry

Paraffin slices were produced from six pairs of oesophageal squamous cell carcinoma tissues and corresponding adjacent normal tissues, followed by deparaffinization in xylene and rehydration in serial dilutions of ethanol. The slices were put into EDTA buffer (pH = 9.0) and the antigen were repaired by high pressure and heating for 30 min. The slices were dripped with endogenous peroxidase blocker, incubated for 10 min at room temperature, and washed with PBS buffer. Then, the slices were sealed for 30 min with 5%BSA. FOXP1 primary antibody (1:150 dilution, BOSTER, M00723‐4) was added and incubated at room temperature for 1 h. The slices were incubated with the secondary antibody for 30 min at room temperature after washing with PBS buffer. The slices were then stained with DAB after cleaning with PBS buffer. Finally, the slices was counterstained with haematoxylin and observed under a microscope.

The score of positively stained cells was applied as follows: 0 (0%), 1 (1%–25%), 2 (26%–50%), 3 (51%–75%) and 4 (76%–100%). The score of intensity staining was defined as follows: 0 for negative, 1 for weak, 2 for moderate and 3 for strong. The total staining score of FOXP1 was calculated by multiplication of the score of intensity staining to the score of positively stained cells.

### 2.6 Tumour immune cell infiltration analysis

We analysed the correlation between FOXP1 expression and immune cell infiltration level in oesophageal cancer using the TIMER database (https://cistrome.shinyapps.io/timer/), and determined the relationship between FOXP1 expression under different somatic copy number alterations (SCNA) levels and the infiltration level of six immune cells in oesophageal cancer, including B cell, CD8 + T cell, CD4 + T cell, macrophage, neurophil and dendritic cell. The TIMER2.0 database (http://timer.cistrome.org/) was applied to investigate the proportion of FOXP1 SCNA in various tumours. The relative abundance of the tumour immune infiltrating cells in oesophageal squamous cell carcinoma tissues was determined by CIBERSORTx database (https://cibersortx.stanford.edu/) using GSE75241 dataset from the GEO database. We used the ‘Pandas’ package in Python to read data and calculate the correlation coefficient matrix, and a correlation heatmap of immune infiltrating cells was drawn using the ‘heatmap’ function in the ‘Seaborn’ package.

### 2.7 Cell culture and plasmids transfection

The oesophageal squamous cell carcinoma cell lines (EC109, KYSE510, EC9706 and TE1) and oesophageal epithelial cell line HEEC were purchased from the Chinese Academy of Sciences Cell Bank. The cells were cultured in DMEM (Hyclone, Logan, UT, USA) supplemented with 10% fetal bovine serum (Hyclone), 100 U/mL penicillin, and 100 U/mL streptomycin in an atmosphere of 5% CO_2_ at 37°C.

We constructed expression vectors encoding FOXP1 and inserted it into the pcDNA3.1 vector (oe‐FOXP1), while the empty pcDNA3.1 plasmids were applied as a negative control (NC). The pcDNA3.1 vector and its corresponding NC for FOXP1 overexpression were synthesized through GenePharma (Shanghai, China). The pcDNA3.1 was linearized by digestion with AflII and XhoI. The forward PCR primer sequence is 5′‐GGCTAGCGTTTAAACTTAAGCCACCATGATGCAAGAATCTGGGAC‐3′ and the reverse PCR primer sequence is 5′‐CGGGCCCTCTAGACTCGAGTCACTCCATGTCCTCGTTTA‐3′. The thermocycling conditions were as follows: 30 cycles of 95°C for 30 s, 56°C for 30 s and 72°C for 60 s. Insertion accuracy was confirmed by Sanger sequencing. The transfection of oesophageal squamous cell carcinoma cell lines was carried out using lipofectamine 2000 (Invitrogen) according to the manufacturer's instructions, and the cells were collected for protein extraction after 72 h.

### 2.8 Western blot analysis

The proteins were extracted by RIPA buffer (Beyotime, Shanghai, China) containing protease and phosphatase inhibitors cocktail (Beyotime). The concentration of proteins was assessed by BCA Protein Assay Kit (Beyotime) according to the manufacturer's instructions. Protein samples were separated by electrophoresis on SDS‐PAGE and transferred to PVDF membranes. The blots were blocked with 5% non‐fat milk for 1 h and incubated with antibodies overnight at 4°C. The membranes were washed with TBST solution three times and then incubated with HRP‐conjugated secondary antibodies at room temperature for 1 h. The Moon Chemiluminescence Reagent kit (Beyotime) was utilized to visualize protein bands.

### 2.9 CCK‐8 assay

The CCK‐8 assay was conducted to determine the effect of FOXP1 on cellular proliferation. Approximately 1 × 10^4^ cells were placed into 96‐well plates with 200 μL DMEM and 10 μL of CCK‐8 solution was added to each well after 24, 48, 72 and 96 h. Following incubation for another 30 min at 37°C, and infinite 200Pro ELISA reader was utilized to analyse the absorbance at 450 nm.

### 2.10 Colony formation assay

The colony formation assay was applied using 60 mm plates in a density of 3000 cells at 37°C in 5% CO_2_ environment for 10 days. The cells were stained with 4% formaldehyde/0.005% gentian violet solution and the colonies were counted under the inverted microscope.

### 2.11 Subcutaneous tumour formation assay

Male BALB/C nude mice (4 weeks old) were purchased from the Shanghai Experimental Animal Centre (Shanghai, China) and kept under specific pathogen‐free conditions. 2 × 10^6^ NC or oe‐FOXP1 EC109 cells were injected subcutaneously into left or right back of each mouse (four mice per group). Tumours were measured every 3 days and the volume of tumour was calculated as *π*/6 × length × width^2^. The mice were euthanized after 30 days, and tumours were harvested and imaged for investigation.

### 2.12 Statistical analysis

The experimental data were presented as the mean ± SD and analysed by GraphPad Prism 6.0 software. The differences were determined by the two‐tailed Student's *t*‐test and Bonferroni correction was performed to correct *p* values in difference comparison between multiple groups. A *p* value of <0.05 was considered statistically significant.

## 3 RESULTS

### 3.1 FOXP1 expression decreases in oesophageal squamous cell carcinoma

**FIGURE 1:**
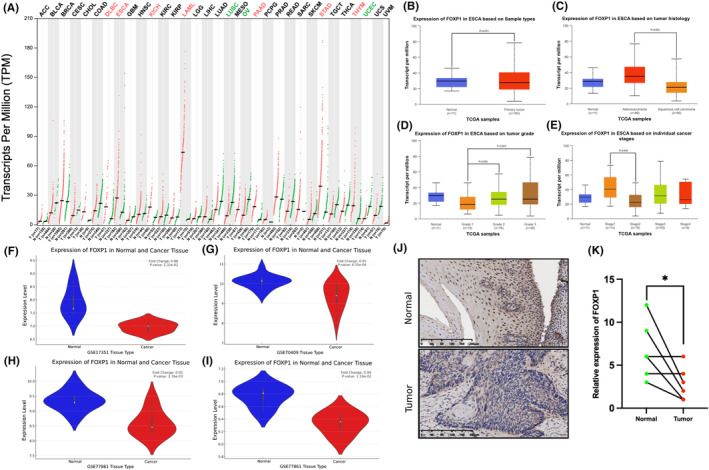
The expression of forkhead box protein 1 (FOXP1) in oesophageal cancer and various tumours. (A) The expression of FOXP1 in various tumours in GEPIA database. (B) The expression of FOXP1 in oesophageal cancer tissues and normal tissues in UALCAN database. (C–E) The expression of FOXP1 in oesophageal cancer tissues depending on pathological type, tumour grade and tumour stage. (F–I) The expression of FOXP1 in oesophageal squamous cell carcinoma in GSE17351, GSE70409, GSE77861 and GSE100942 datasets. (J–K) The expression of FOXP1 in oesophageal squamous cell carcinoma tissues and adjacent normal tissues. *p < 0.05.

We first compared the expression of FOXP1 in various tumours through the GEPIA database, which containing data from TCGA and GTEx programs. The results showed that the mRNA expression of FOXP1 increases significantly in DLBC, ESCA, KICH, LAML, PAAD, STAD and THYM, while it decreases in LUSC, OV and UCEC (Figure 1A). By analysing TCGA data through the UALCAN database, we found that there was no significant difference in FOXP1 expression between oesophageal cancer and normal tissues (Figure 1B). However, further analysis showed that the expression level of FOXP1 in oesophageal squamous cell carcinoma was significantly lower than that in oesophageal adenocarcinoma (Figure 1C), and FOXP1 expression varies in different tumour stages and grades (Figure 1D,E). We further analysed the GEO data and verified that the mRNA expression level of FOXP1 significantly reduces in oesophageal squamous cell carcinoma (Figure 1F–I). Furthermore, we verified that the expression of FOXP1 is significantly reduced in oesophageal squamous cell carcinoma tissues compared to normal tissues through immunohistochemistry in Figure 1J,K.

### 3.2 High FOXP1 expression is related with a better prognosis

**FIGURE 2:**
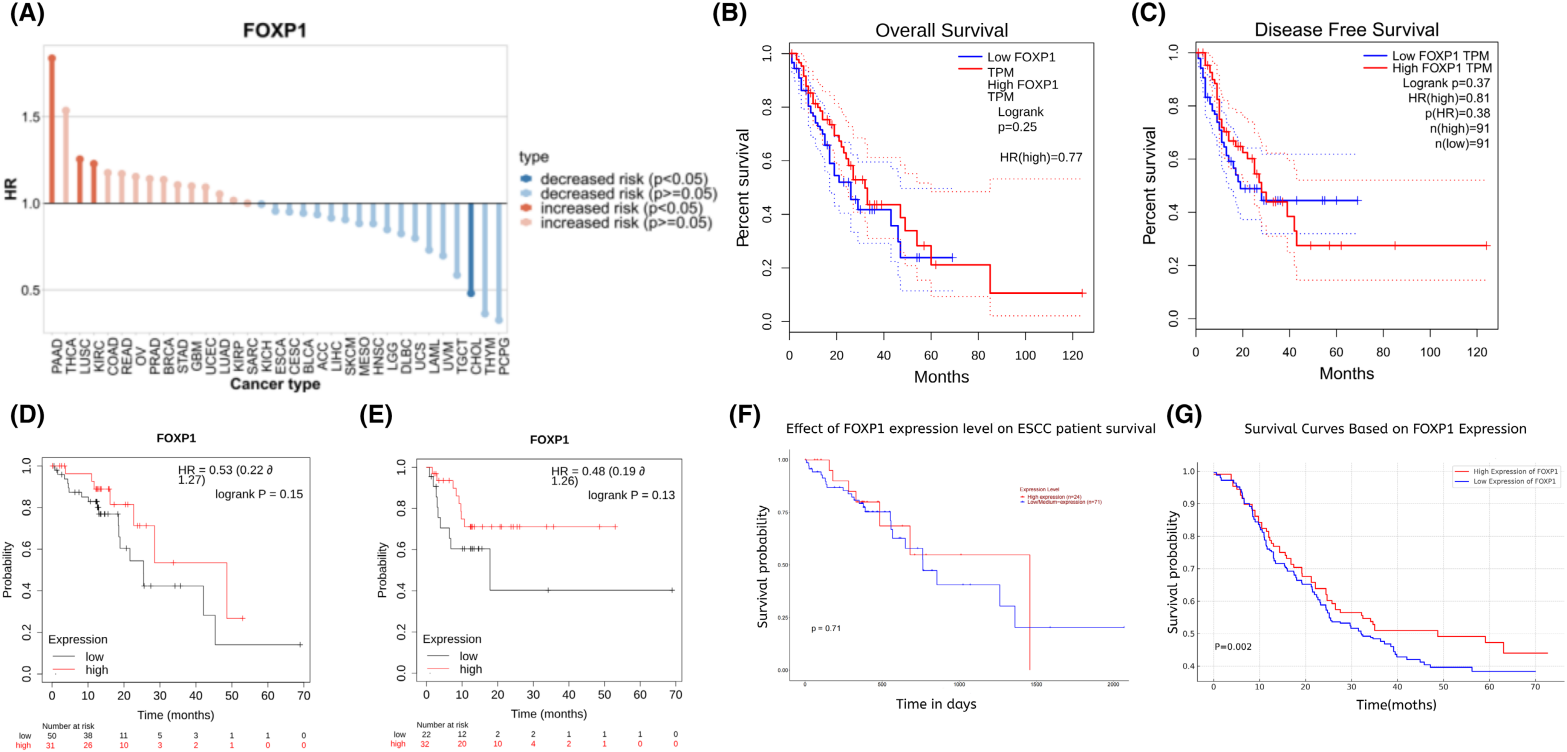
High expression of forkhead box protein 1 (FOXP1) indicates a better survival prognosis in oesophageal cancer. (A) The correlation between expression of FOXP1 and overall survival prognosis in different tumours in TISCH2 database. (B) Kaplan–Meier survival curves of overall survival for oesophageal cancer patients with high and low expression of FOXP1 in GEPIA database. (C) Kaplan–Meier survival curves of disease free survival for oesophageal cancer patients with high and low expression of FOXP1 in GEPIA database. (D) Kaplan–Meier survival curves of overall survival for oesophageal cancer patients with high and low expression of FOXP1 in KM plotter database. (E) Kaplan–Meier survival curves of disease free survival for oesophageal cancer patients with high and low expression of FOXP1 in KM plotter database. (F) Kaplan–Meier survival curves of overall survival for oesophageal squamous cell carcinoma patients with high and low expression of FOXP1 in KM plotter database. (G) Kaplan–Meier survival curves of overall survival for oesophageal squamous cell carcinoma patients with high and low expression of FOXP1 in GSE53625 dataset.

As shown in Figure 2A, FOXP1 predicts different overall survival outcomes in different tumours, and high expression of FOXP1 in oesophageal cancer may be associated with a better prognosis. The results from the GEPIA and KM Plotter databases showed that oesophageal cancer patients with high FOXP1 expression have better overall survival and disease‐free survival despite the difference is not significant (Figure 2B–F), which may be related to the insufficient sample size of oesophageal cancer patients in these databases. We further determined that high FOXP1 expression indicates a better prognosis in oesophageal squamous cell carcinoma in the GEO database (Figure 2G).

### 3.3 Identification of differentially expressed genes in high and low expression of FOXP1 cohorts

**FIGURE 3:**
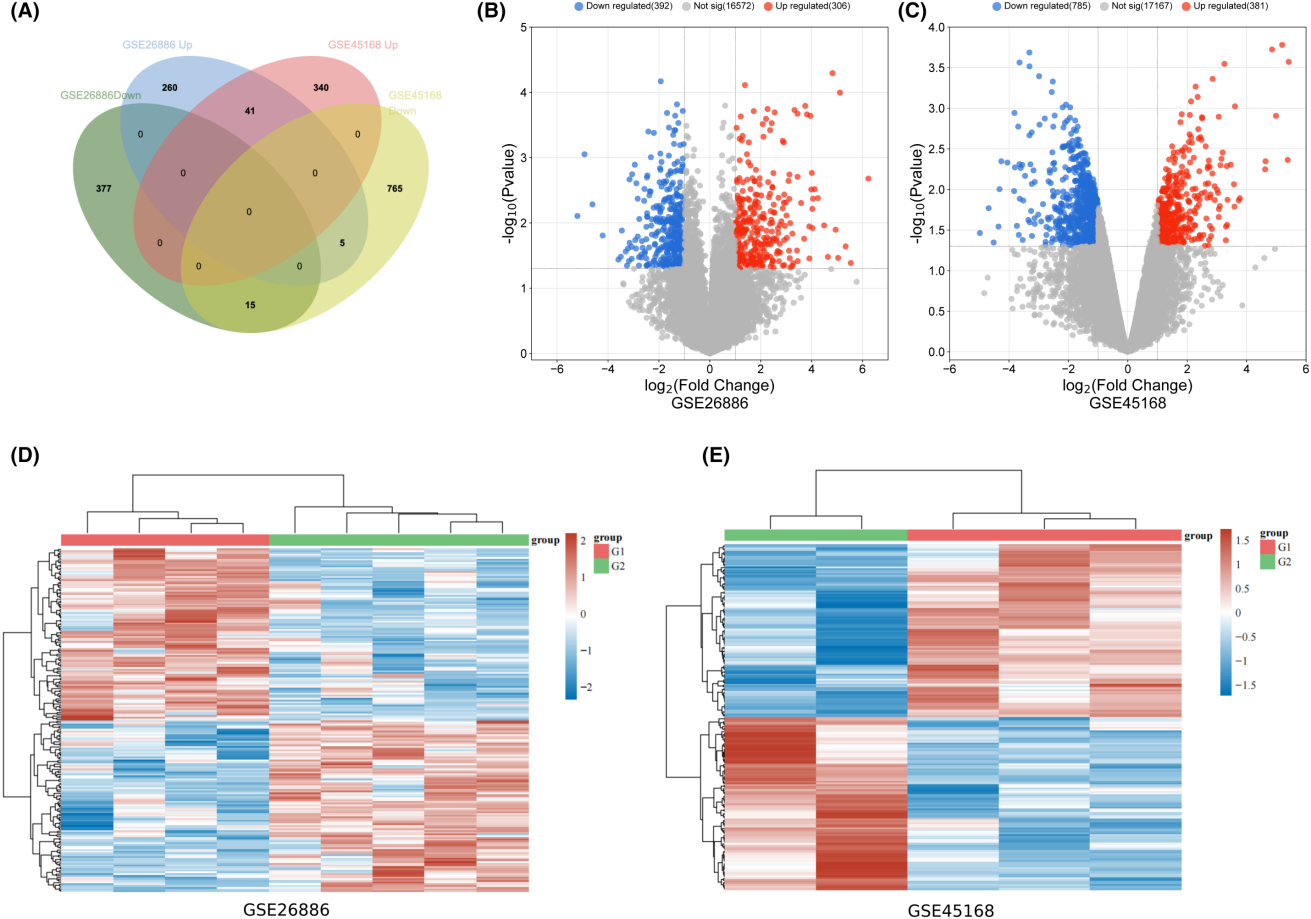
Identification of differential expression genes in high and low expression of forkhead box protein 1 (FOXP1) cohorts in oesophageal squamous cell carcinoma. (A) Venn diagram showing the intersection of differential expression genes in GSE26886 and GSE45168 datasets. (B, C) Volcano plots showing the differential expression genes in GSE26886 and GSE45168 datasets. (D, E) Cluster heatmaps of genes in GSE26886 and GSE45168 datasets.

As shown in Figure 3A, we compared the differentially expressed genes in oesophageal squamous cell carcinoma tissues with high and low expression of FOXP1 using the GSE26886 and GSE45168 datasets. There are 306 upregulated genes and 392 downregulated genes identified in the GSE26886 dataset, while 381 upregulated genes and 785 downregulated genes were determined in the GSE45168 dataset. Among them, 41 upregulated genes and 15 downregulated genes express significantly differentially in both datasets. The differentially expressed genes volcano plots were shown in Figure 3B,C, and the cluster heatmaps were shown in Figure 3D,E.

### 3.4 FOXP1 functional analysis and interaction network construction

**FIGURE 4:**
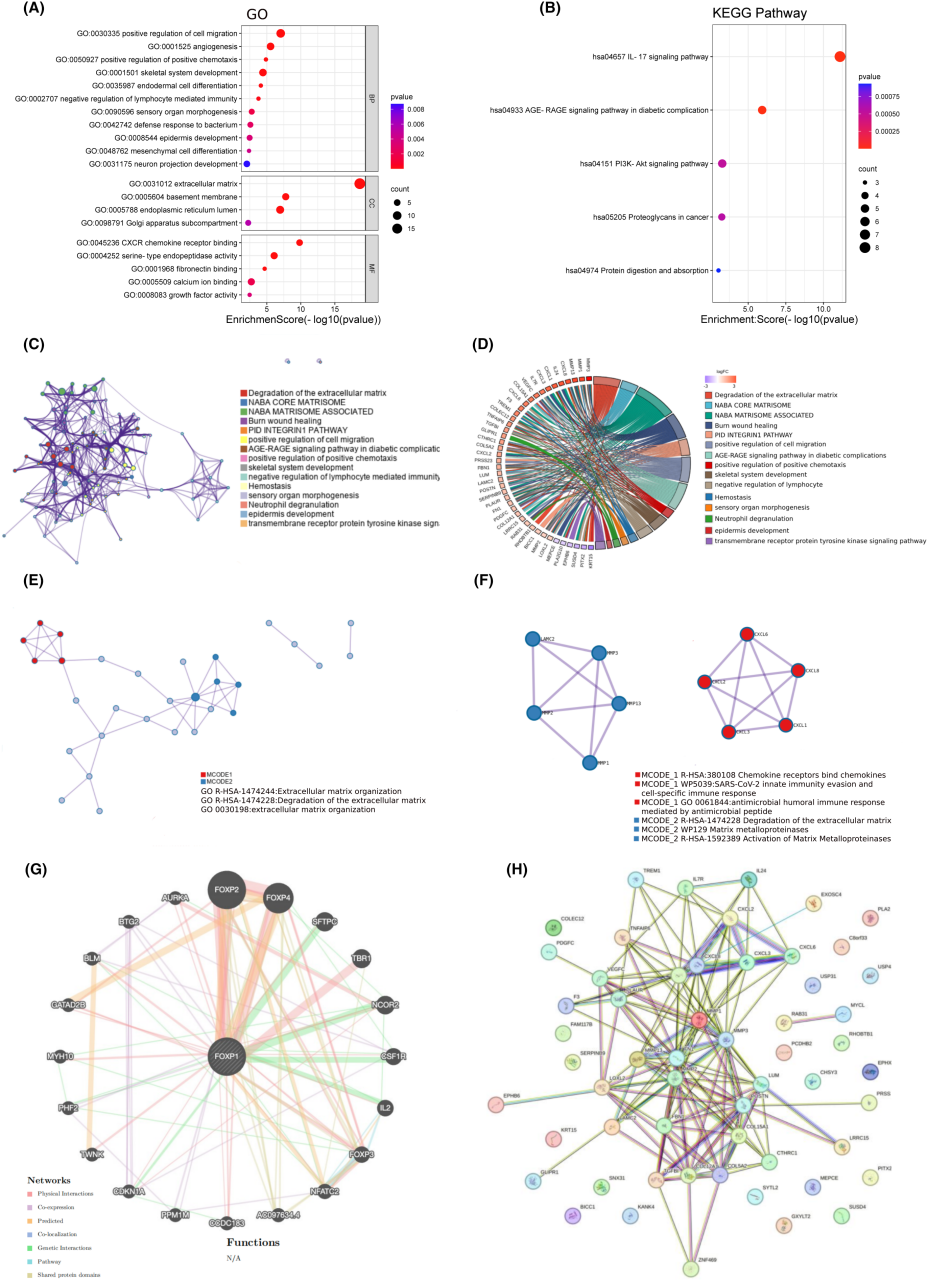
Forkhead box protein 1 (FOXP1) functional analysis and interaction network construction. (A) Gene ontology enrichment analysis for differential expression genes. (B) KEGG enrichment analysis for differential expression genes. (C) Network of enriched terms in metascape database. (D) The cord diagram of the correspondence between differential expression genes and signalling pathways. (E, F) Protein–protein interaction (PPI) network and MCODE components identified in differential expression genes in Metascape database. (G) The gene co‐expression network for FOXP1 in GeneMANIA database. (H) PPI network of differential expression genes in STRING database.

To determine the potential molecular mechanism of FOXP1 affecting the progression of oesophageal squamous cell carcinoma, we performed GO and KEGG functional enrichment analysis on 41 upregulated genes and 15 downregulated genes. The results showed that these differentially expressed genes were involved in regulation of cell migration, angiogenesis, extracellular matrix, CXCR chemokine receptor binding and other biological processes, as well as IL‐17 signalling pathway, AGE‐RACE signalling pathway in diabetic complication, PI3K‐Akt signalling pathway, Proteoglycans in cancer, protein digestion and absorption signalling pathways (Figure 4A,B). We found that these genes are also involved in biological processes related to tumour progression in Metascape database, such as regulation of cell migration and regulation of lymphocyte mediated immunity (Figure 4C). The cord diagram of the correspondence between differentially expressed genes and signalling pathways was shown in Figure 4D, and two functional modules with close protein expression relationships were identified by the MCODE algorithm (Figure 4E,F). Figure 4G showed that FOXP1 co‐expresses with genes such as FOXP2, FOXP4, SFTPC, TBR1, NCOR2, CSF1R, IL2 and FOXP3. We further constructed a PPI network using the STRING database as shown in Figure 4H.

### 3.5 Association of FOXP1 with the tumour immune microenvironment

**FIGURE 5:**
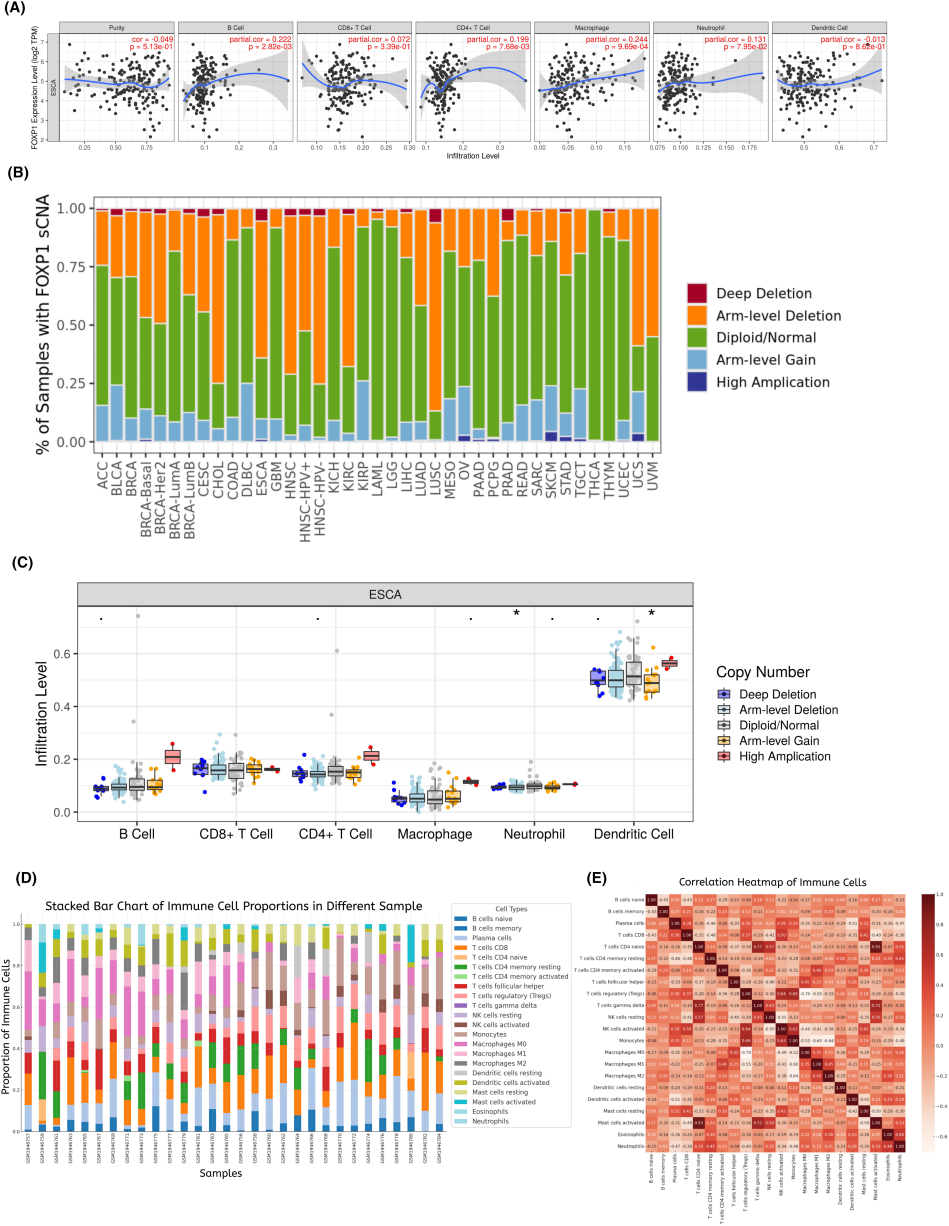
Association of forkhead box protein 1 (FOXP1) with the tumour immune microenvironment in oesophageal cancer. (A) The association of FOXP1 expression and tumour immune infiltration cells level in oesophageal cancer in TIMER database. (B) The relative proportion of different somatic copy number alterations (SCNA) states of FOXP1 for various TCGA tumour types in TIMER2.0 database. (C) The relationship between different SCNA states of FOXP1 and infiltration level of six types of immune cells in oesophageal cancer. *p < 0.05. (D) The relative proportion of the infiltration levels of 22 immune infiltrating cells in oesophageal squamous cell carcinoma based on GSE75241 dataset. (E) The immune cells correlation heatmap in oesophageal squamous cell carcinoma based on GSE75241 dataset.

As shown in Figure 5A, FOXP1 expression is significantly positively correlated with B cells (*R* = 0.222, *p* = 2.82e‐03), CD4+ T cells (*R* = 0.199, *p* = 7.68e‐0.3) and macrophage (*R* = 0.244, *p* = 9.69e‐04) infiltration level. Figure 5B showed the relative proportion of different SCNA states of FOXP1 for all TCGA tumour types. In oesophageal cancer, arm‐level deletion is the main type of SCNA of FOXP1. The relationship between different SCNA states of FOXP1 and infiltration level of six types of immune cells in oesophageal cancer was shown in Figure 5C. The arm‐level deletion decreases in neutrophil and arm‐level gain reduces in dendritic cells significantly. Based on the GSE75241 dataset, we analysed the infiltration levels of 22 immune infiltrating cells in oesophageal squamous cell carcinoma (Figure 5D). We further constructed an immune cells correlation heatmap. As shown in Figure 5E, there is a significant positive correlation between T‐cells gamma delta and T cells CD4 naive (*R* = 0.77), mast cells activated and T cells CD4 naive (*R* = 0.93), as well as mast cells activated and T‐cells gamma delta (*R* = 0.72).

### 3.6 FOXP1 inhibits proliferation of oesophageal squamous cell carcinoma cells

**FIGURE 6:**
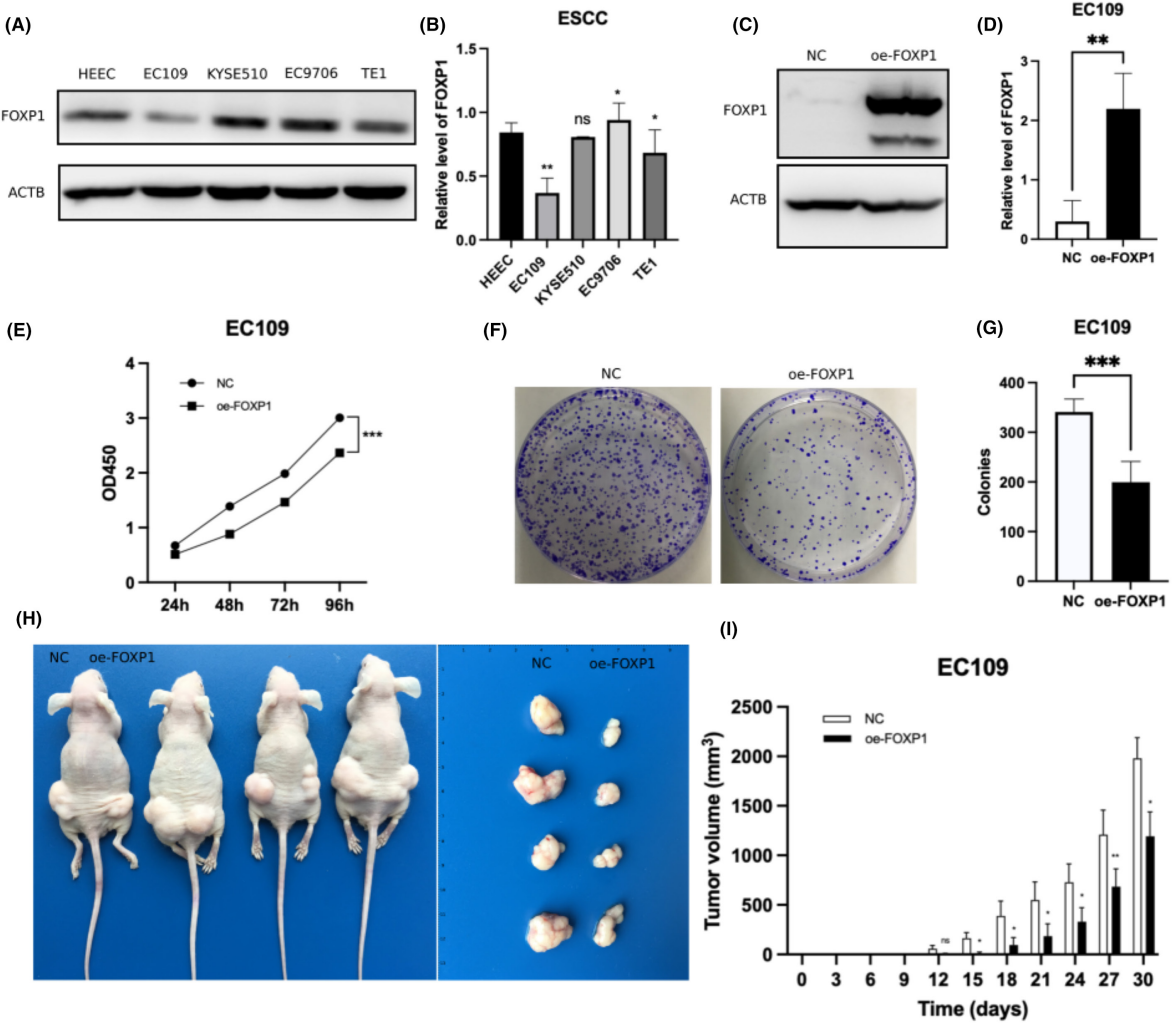
Forkhead box protein 1 (FOXP1) inhibits proliferation of oesophageal squamous cell carcinoma cells. (A, B) Western blot assay confirmed the expression levels of FOXP1 protein in different oesophageal squamous cell carcinoma cell lines and oesophageal epithelial cells. *p < 0.05, **p < 0.01, ns *p* > 0.05. (C, D) Western blot assay confirmed the transfection efficiency of overexpression of FOXP1 in EC109 cells. **p < 0.01. (E) The CCK‐8 assay showed overexpression of FOXP1 inhibits the proliferation of EC109 cells. ***p < 0.001. (F, G) The colony formation assay showed overexpression of FOXP1 inhibits the colony forming ability of EC109 cells. (H, I) The subcutaneous tumour formation assay showed overexpression of FOXP1 inhibits the ability of tumorigenicity of EC109 cells in vivo. *p < 0.05, **p < 0.01, ns *p* > 0.05.

We first compared the expression levels of FOXP1 protein in different oesophageal squamous cell carcinoma cell lines and oesophageal epithelial cells. As shown in Figure 6A,B, FOXP1 protein reduces significantly in EC109 and TE1 cells, while it increases in EC9706 cells. Then, we chose EC109 cells for further research. We overexpressed FOXP1 in the EC109 oesophageal squamous cell carcinoma cell line, and Western blot assay confirmed the transfection efficiency (Figure 6C,D). Through the CCK‐8 assay, we found that overexpression of FOXP1 inhibits the proliferation of oesophageal squamous cell carcinoma cells (Figure 6E). The colony formation assay revealed that the colony forming ability of oesophageal squamous cell carcinoma cells significantly decreases after overexpression of FOXP1 (Figure 6F,G). We further validated the inhibitory effect of FOXP1 on the proliferation of oesophageal squamous cell carcinoma cells in vivo through subcutaneous tumour formation assay (Figure 6H,I). These results revealed that overexpression of FOXP1 inhibits proliferation of oesophageal squamous cell carcinoma cells.

## 4 DISCUSSION

Treatment for oesophageal squamous cell carcinoma has advanced significantly in recent years, however, the therapeutic effect is still not sufficient. Patients have a 5‐year overall survival rate of 30%,^28^, ^29^ and reliable biomarkers for prognosis prediction are scarce. The mechanism of occurrence and progression of oesophageal squamous cell carcinoma is poorly understood, and researches on novel biological targets for tumour treatment are still limited. FOXP1 is an important transcription factor of the forkhead box protein family, which has differential expression in various tumour tissues. It highly expresses in diffuse large B‐cell lymphoma^30^ and low expression of FOXP1 is found in extrahepatic cholangiocarcinoma.^31^ In our study, FOXP1 expression level in oesophageal squamous cell carcinoma tissue was determined by public database analysis and experiments. Although the GEPIA database, which contains TCGA and GTEx data, indicated that FOXP1 expression is high in oesophageal cancer, results from the UALCAN database indicated that FOXP1 expression is high in oesophageal adenocarcinoma, while low expression of FOXP1 is observed in oesophageal squamous cell carcinoma. Additionally, the GEO database and our immunohistochemical staining results verified that oesophageal squamous cell carcinoma exhibits low expression of FOXP1 compared with normal tissues. These results suggest that FOXP1 plays different biological roles in oesophageal squamous cell carcinoma and oesophageal adenocarcinoma. Wei et al.^32^ found that a better prognosis for oesophageal squamous cell carcinoma patients is predicted by elevated nuclear expression of FOXP1. Our study also revealed that oesophageal squamous cell carcinoma patients with high FOXP1 expression had a better prognosis, despite certain datasets did not demonstrate statistically significant differences. These findings imply that FOXP1 may be involved in the proliferation of tumour cells in oesophageal squamous cell carcinoma.

Through examining the differentially expressed genes in oesophageal squamous cell carcinoma tissues with high and low FOXP1 expression in the GSE26886 and GSE45168 datasets, we identified 41 significantly upregulated genes and 15 significantly downregulated genes. These genes are potentially regulated by the transcription factor FOXP1. We further conducted GO and KEGG enrichment analysis on these genes and found that they are involved in biological processes and signalling pathways related to tumour progression, such as extracellular matrix structural constraint, IL‐17 signalling pathway, PI3K‐Akt signalling pathway, regulation of cell migration, regulation of lymphocyte mediated immunity, and so on. Studies have shown that FOXP1 participates in the occurrence and development of various tumours through different molecular mechanisms. FOXP1 is identified as a negative regulator of immune responses by regulating expression of cytokine and chemokine in breast cancer,^8^ which is consistent with the results obtained from our enrichment analysis. Additionally, Chen et al.^33^ revealed that NAT10/ac4C/FOXP1 axis promotes malignant progression and immunosuppression of cervical cancer. Li et al.^34^ found that FOXP1 promote the progression of osteosarcoma by repressing the transcription of P21 and RB protein and directly interacting p53 to inhibit its activity. Chen et al.^35^ pointed out that FOXP1 transcriptionally activates GINS1 expression to promote diffuse large B‐cell lymphoma development. In oesophageal squamous cell carcinoma, exosome lncRNA FAM225A upregulates the expression of NETO2 and FOXP1 by absorbing miR‐206, participating in progression and angiogenesis of tumor.^36^ We outlined the biological processes and molecular mechanisms of FOXP1 in oesophageal squamous cell carcinoma through GO and KEGG functional enrichment, gene co‐expression analysis, and PPI network construction. Further investigations are warrant to explore and verify these potential mechanisms, which FOXP1 involves in oesophageal squamous cell carcinoma.

FOXP1 has different biological effects in different cancers playing a role in promoting or suppressing tumour. In pancreatic cancer, FOXP1 inhibits growth of tumour cell by transcriptionally regulating expression of IRF1.^37^ In lung adenocarcinoma, FOXP1 is also determined as a tumour suppresser gene by inhibiting chemokine signalling pathways.^21^ In multiple myeloma^38^ and diffuse large B‐cell lymphoma,^16^ FOXP1 is found to promote tumorigenesis. In our research, cytological experiments were conducted to reveal that the proliferation ability, colony forming ability and in vivo tumorigenesis ability of EC109 oesophageal squamous cell carcinoma cells with overexpression of FOXP1 are significantly downregulated. These results suggest that FOXP1 serves as a tumour suppressor in oesophageal squamous cell carcinoma and can be a potential biological target for the treatment of tumour.

Tumour immune infiltrating cells are involved in tumour development, angiogenesis, tumour cell growth and metastasis. The progression of tumours may be caused by immune escape and inadequate host immune surveillance. There have been reports indicating that immune infiltration is involved in the progression of various digestive system tumours and can serve as a prognostic factor. Jin et al.^39^ explored the differences of immune infiltration cells in gastric cancer and they found that activated CD4+ memory T cells and Tfh are related to worse overall survival and progression‐free survival, while that naive B cells are the reverse for progression‐free survival. Wang et al.^40^ found a new subset of B cells (LARS B) with a TGF‐β1‐dominant regulatory feature and they play a role in inhibition of anti‐tumour immune effects in colorectal cancer. The ceRNA network controls a wide range of cancer‐related hallmarks, and Wang et al.^41^ identified certain ce‐RNA‐related protein coding genes involved in various biological processes, including avoiding immune destruction, which promote progression of oesophageal squamous cell carcinoma. Therefore, exploring factors regulating tumour immune infiltration levels is of importance. Specific lncRNAs, proteins, and related pathways have been confirmed to be involved in the development of oesophageal squamous cell carcinoma and the regulation of tumour immune infiltration.^42^, ^43^, ^44^, ^45^ Luo et al.^46^ also pointed out metabolic alteration mediated by hypoxia and TP53 mutation is related to regulation of tumour immune microenvironment across cancer types. Tumour immune infiltrating cells are related to the development of digestive system tumours. Therefore, we further investigated whether FOXP1 is a potential regulatory factor for immune infiltration levels in oesophageal squamous cell carcinoma.

Through the analysis of TIMER database, we found that FOXP1 expression is significantly positively correlated with B cell, CD4 + T cell, and macrophage expression level in oesophageal cancer. Seung et al.^47^ reported that in addition to assisting CD8+ T cells in killing breast cancer cells, CD4 + T cells also have direct anti‐tumour effects. The CD4+ T cells are revealed to be able to kill autologous tumour cells in a MHC class II‐dependent manner.^48^ The antibodies produced by B cells can directly kill tumour cells through antibody dependent cytotoxicity (ADCC) and phagocytosis, and B cells can also serve as a dedicated antigen presenting cell (APC) to activate T cells to inhibit cancer.^49^, ^50^, ^51^ Therefore, our study suggests that the reduced expression of FOXP1 in oesophageal squamous cell carcinoma may lead to downregulation of CD4+ T cells and B cells, promoting immune escape of tumour cells and progression of oesophageal cancer. NAT10/ac4C/FOXP1 axis was reported to facilitate immunosuppression and overexpression of FOXP1 was found to be positively related to the infiltration of activated CD4+ T cells and Tregs in cervical cancer,^33^ which supports our hypothesis. In breast cancer, the expression of FOXP1 is positively correlated with the infiltration of CD4 + T cells, CD8 + T cells, neutrophils and macrophages, but different from our findings, the expression of FOXP1 is negatively correlated with B cells.^52^ We speculated that FOXP1 may regulate different immune infiltrating cells depending on different tumours. In addition, the differentially expressed genes identified in our research contain chemokines, such as CXCL1, CXCL2, CXCL3, CXCL6 and CXCL8, which are associated with recruitment of dendritic cells, natural killer cells and CD8+ T cells.^53^, ^54^, ^55^ This further indicates that FOXP1 may participate in the regulation of tumour immune infiltrating cells by regulating the expression of chemokine family related proteins, thereby inhibiting the growth of oesophageal squamous cell carcinoma cells. More experiments are needed to explore the mechanism of FOXP1 regulating tumour immune infiltrating cells.

## 5 CONCLUSION

Our research elucidated low expression of FOXP1 in oesophageal squamous cell carcinoma and it can serve as a prognostic predictor. The inhibitory effect of FOXP1 on tumour growth in oesophageal squamous cell carcinoma was experimentally verified. Potential biological processes and signalling pathways involved in the incidence and development of oesophageal squamous cell carcinoma were also revealed. FOXP1 has the potential to regulate tumour immune infiltrating cells and play a role in anti‐tumour immunity. Consequently, FOXP1 may function as a novel biological target for tumour therapy as well as a biomarker for predicting the prognosis of oesophageal squamous cell carcinoma.

## AUTHOR CONTRIBUTIONS

**Guanzhi Ye:** Investigation (equal); methodology (equal); writing – original draft (lead); writing – review and editing (equal). **Gaojian Pan:** Investigation (equal); methodology (equal); visualization (equal). **Xiaolei Zhu:** Formal analysis (equal); resources (equal). **Ning Li:** Investigation (equal). **Hongming Liu:** Visualization (equal). **Guojun Geng:** Data curation (lead); project administration (equal); validation (equal). **Jie Jiang:** Conceptualization (lead); supervision (lead).

## FUNDING INFORMATION

This study was supported by Natural Science Foundation of Fujian Province (no. 2022 J05301 and 2021 J05284), and Xiamen Municipal Bureau of Science and Technology (no. 3502Z20214ZD3011).

## CONFLICT OF INTEREST STATEMENT

The authors declare they have no conflicts of interest.

## Data Availability

The data that support the findings of this study are available from the corresponding author upon reasonable request.
